# Analgesic Effects and Impairment in Locomotor Activity Induced by Cannabinoid/Opioid Combinations in Rat Models of Chronic Pain

**DOI:** 10.3390/brainsci10080523

**Published:** 2020-08-06

**Authors:** Mohammad Alsalem, Ahmad Altarifi, Mansour Haddad, Belal Azab, Heba Kalbouneh, Amer Imraish, Tareq Saleh, Khalid El-Salem

**Affiliations:** 1 Faculty of Medicine, The University of Jordan, Amman 11942, Jordan; azab.belalm@gmail.com (B.A.); heba.kalbouneh@gmail.com (H.K.); 2 Faculty of Medicine, Jordan University of Science and Technology, Irbid 22110, Jordan; aaaltarifi@just.edu.jo (A.A.); khalidelsalem@hotmail.com (K.E.-S.); 3 Faculty of Pharmacy, Philadelphia University, Amman 19392, Jordan; dr.man.haddad@gmail.com; 4 Faculty of Science, The University of Jordan, Amman 11942, Jordan; a.imraish@ju.edu.jo; 5 Faculty of Medicine, The Hashemite University, Zarqa 13133, Jordan; tareq@hu.edu.jo

**Keywords:** chronic pain, Complete Freund’s Adjuvant (CFA), streptozotocin (STZ), von Frey filament test, morphine, tramadol, WIN55212, HU210, open field test

## Abstract

Both opioids and cannabinoids have well-known antinociceptive effects in different animal models of chronic pain. However, unwanted side effects limit their use. The aim of this study is to evaluate the antinociceptive effect of combining synthetic cannabinoids with subtherapeutic doses of opioids, and to evaluate the effects of these drugs/combinations on rat’s locomotor activity. Intra-plantar injection of Complete Freund’s Adjuvant (CFA) into the left hindpaw and intraperitoneal injection of streptozotocin (STZ) were used to induce inflammatory and diabetic neuropathic pain in adult male Sprague-Dawley rats, respectively. Von Frey filaments were used to assess the antinociceptive effects of opioids (morphine and tramadol) and the synthetic cannabinoids (HU210 and WIN55212) or their combinations on CFA and STZ-induced mechanical allodynia. Open field test was used to evaluate the effect of these drugs or their combinations on locomotion. HU210 and WIN55212 did not produce significant antinociceptive effect on inflammatory pain while only the maximal dose of HU210 (1 mg/kg) was effective in neuropathic pain. Only the maximal doses of morphine (3.2 mg/kg) and tramadol (10 mg/kg) had significant anti-allodynic effects in both models. Tramadol (1 mg/kg) enhanced the antinociceptive effects of WIN55212 but not HU210 in neuropathic pain with no effect on inflammatory pain. However, in open field test, the aforementioned combination did not change tramadol-induced depression of locomotion. Tramadol and WIN55212 combination produces antinociceptive effects in neuropathic but not inflammatory pain at low doses with no additional risk of locomotor impairment, which may be useful in clinical practice.

## 1. Introduction

Chronic pain is a major healthcare problem that imposes huge social and economic burden all over the world. Most of the currently used analgesics such as non-steroidal anti-inflammatory drugs and opioids have limited efficacy and/or accompanied by unwanted side effects [1,2]. Consequently, the need for therapeutic agents capable of alleviating pain without impairing normal functions remains largely unmet. Considering the physiological role of cannabinoid receptors (CB1 and CB2) in controlling multiple responses including pain, cannabinoids exert analgesic effects in various models of chronic pain [3]. In clinical studies, cannabinoids have promising analgesic properties in various clinical conditions. However, the wide distribution of cannabinoid and opioid receptors in the brain underpins both the therapeutic effects of cannabinoids and opioid such as analgesia, as well as their unwanted side effects such as hypomotility, nausea, sedation, constipation, respiratory depression and risk of developing tolerance and addiction [4,5].

Although new targets and receptors are continuously being identified to control pain, the development of effective agents from these targets probably will take a significant time and effort. For the time being, using a combination of already known compounds with different mechanisms of action could achieve therapeutic effects at lower concentrations of each compound and potentially minimize the intensity and occurrence of unwanted side effects. Previous studies have reported promising synergistic effects between cannabinoids and opioids in different pain models [6,7,8]. Surprisingly, a few studies evaluated the side effects of these combinations, especially effects on general behaviors, such as locomotion.

Cannabinoids and opioids share very similar signal transduction properties, and they are co-localized in the presynaptic terminal in areas involved in pain processing, and co-localized in various CNS regions, such as periaqueductal gray neurons (PAG), dorsal horn, raphe nuclei, nucleus accumbens and forebrain [9,10]. These data suggest that cannabinoid and opioid receptors could work together to produce analgesia, and the activation of one receptor could interfere or modulate the activity of the other. Indeed, the literature review indicates the presence of functional interactions between opioid and cannabinoid receptors, and therefore, a combination therapy of these drugs is strongly believed to effectively alleviate chronic pain. For example, rimonabant, a CB1 receptor antagonist, was found to weaken morphine’s antinociceptive effects [11,12], while administration of opioid antagonists blocked the effects of Δ9-THC [13]. In humans, small-scale clinical trials established that after vaporized or oral exposure to cannabis, the antinociceptive effects of opioids were significantly enhanced [7,8,14]; however, it was not shown whether they had synergistic or additive effects. Multiple isobolographic studies showed synergism between morphine and Δ9-THC in animal models of acute and inflammatory pain [15,16,17]. This antinociceptive synergy could be mediated by either CB1 or CB2 receptors; depending on the pain model used. Furthermore, WIN55212, a synthetic cannabinoid receptor agonist, produced CB1-mediated synergystic antinociception when co-administered with morphine in the formalin test, but not the carrageenan test [18]. Contrarily, GP1a, a CB2 receptor agonist, enhanced analgesia in the carrageenan test when administered with morphine [18]. Another isobolographic study showed that co-administration of WIN55212 with morphine had a synergistic anti-allodynic effect while only causing an additive effect on locomotor disruption [19]. Further, a previous study supported these findings in which co-administration of μ-opioid and CB2 receptor agonists synergistically reduced the nociceptive behaviour induced in different models of pain, while attenuating the undesired side-effects [20]. The aforementioned studies, although provide a compelling evidence that the co-administration opioid and cannabinoid agonists could be useful clinically; few of them evaluated their effects on locomotor activity. From our point of view, effects of previously tested combinations may be compound specific, therefore a correct pairing of the most effective combinations is required. Therefore, the aim of the current study is to systematically assess the antinociceptive effects of certain synthetic cannabinoids (HU210 and WIN55212) when co-administered with subtherapeutic doses of opioid agonists (morphine and tramadol) on both STZ-induced diabetic neuropathy and CFA-induced inflammatory pain models. Moreover, the effect of those cannabinoid/opioid combinations will be evaluated on locomtion by using the open field test.

## 2. Materials and Methods

### 2.1. Animals

Behavioral investigation was conducted using adult male Sprague-Dawley rats (180–250 g, obtained from the Animal House Unit, The University of Jordan). Rats housing and husbandry was done in The University of Jordan Animal House Unit; in a temperature-regulated environment 22 ± 1 °C under a 12 h: 12 h/light: dark cycle. Total number of rats used in this study 220. Experimental procedures were approved by the scientific research committee at the University of Jordan, approval number (19\2018\321), date 14\08\2018. All experiments were carried-out according to the Animal (Scientific Procedure) Act 1986 and International Association for the Study of Pain guidelines.

### 2.2. Induction of Inflammatory Pain Model

In order to induce inflammatory pain, complete Freund’s adjuvant (CFA; 50% in saline, with 5 mg/mL heat-killed *Mycobacterium tuberculosis*, 0.1 mL) or vehicle were injected into the plantar surface of the left hindpaw of each rat. The volume of vehicle administered to naive rats was equal to that of CFA; 0.1 mL of saline [21,22]. After the determination of the baseline nociceptive threshold, testing was performed every other day for 21 days following the CFA injection.

### 2.3. Induction of Diabetic Neuropathy Pain Model

To induce diabetes, rats received intraperitoneal (I.P.) injections of STZ (35 mg/kg, Tocris Bioscience, Bristol, UK) or vehicle (0.1 M citrate buffer pH 4.6). These injections were performed under brief isoflurane anesthesia. Baseline reading of mechanical allodynia, blood glucose concentrations and body weight were taken before the injections, and then tested every week for 8 weeks [23]. In order to confirm diabetes, Accu-Check performa (Roche, Grenzach-Wyhlen, Germany) was used to measure the glucose concentration in blood samples collected from the tail vein. Three days following the STZ injections, rats with glucose levels higher than 200 mg/dl were regarded as diabetic.

### 2.4. Assessment of Mechanical Allodynia

Mechanical allodynia was determined using the von Frey filament test. Each rat was placed in a plastic compartment and behavioral settlement was permitted for at least 25 min; until cage adaptation and major grooming activities stopped. The plastic cages possess wire mesh bottom allowing for sufficient access to the rats’ paws. To evaluate the withdrawal threshold, the “up-down” approach was used by applying the von Frey filaments (2–15 g, with logarithmically incremental stiffness; Bioseb, Vitrolles, France) to the mid-plantar surface of each rat’s left hindpaw. The von Frey hair was held for about 6–8 s perpendicularly to the plantar surface of the left hind paw [24,25]. Data were presented in grams as paw withdrawal thresholds (PWT).

### 2.5. Open Field Test

To evaluate the effects of different drugs and combinations on locomotor activity, a computed animal activity system (Opto-M4, Columbus Instruments, Columbus, OH, USA) was used. This open field system consists of a 45 × 25 × 20 cm arena with 2 horizontal planes of detector-emitter pairs across the width of the arena; positioned 5 cm and 10 cm above the cage floor. Each horizontal plane is monitored by 16 infrared beams spaced 2.5 cm apart. Animals were placed individually 60 min after drug injections. For 20 min, each rat’s movement through the infrared beams was calculated as the total numbers of beam interruptions every 5 min [26]. This allows the system to continuously monitor the horizontal (X axis) and vertical (Z axis) activity. Horizontal activity is represented as % X total counts. % X total counts = (X total counts after drug application/baseline X total counts) × 100. X total counts register a count every time an infrared beam is broken in the lower plane, which reflects the overall locomotor activity and the repetitive small scale movements such as scratching and grooming. % Z total counts (Z total counts after drug application/baseline Z total counts × 100) were used to represent the vertical activity. Z total counts register a count every time an infrared beam is broken in the upper plane and is utilized to detect rearing or standing on the hind paws. Rats first received intraperitoneal injections of different drugs or combination. Their effect on locomotion was then evaluated 1 h post-injection. The observer was blinded to treatment during all the behavioral experiments.

### 2.6. Pharmacological Treatments

The effects of different drugs on both the CFA and STZ-induced nociceptive behavior were assessed: HU210 (0.1, 0.32 and 1 mg/kg), WIN55212 (0.1, 0.32 and 1 mg/kg), morphine (0.32, 1 and 3.2 mg/kg) and tramadol (1, 3.2 and 10 mg/kg) were administered through intraperitoneal (I.P.) injections (0.5 mL, *n* = 8 rats/group). von Frey filament test were performed 1 h after drug/combination administration.

To assess the effect of opioids/cannabinoid combinations on CFA and STZ-induced mechanical allodynia, a subtherapeutic dose of morphine (0.32 mg/kg) was co-administered with the different doses of HU210 (0.1, 0.32 and 1 mg/kg) or WIN55212 (0.1, 0.32 and 1 mg/kg). Similarly, a subtherapeutic dose of tramodol (1 mg/kg) was co-administered with the different doses of HU210 (0.1, 0.32 and 1 mg/kg) or WIN55212 (0.1, 0.32 and 1 mg/kg).

In the CFA pain model, drug injections were administered (single dose) at days 3–9 post-CFA injection using Latin square design (Table 1); when mechanical allodynia was fully developed. In the STZ pain model, injections were administered (single dose) at days 1–7 in week 4 post-STZ injection using Latin square design (Table 2); when mechanical allodynia became prominent. The observer was blinded to treatment during all the behavioral experiments. The effects of different drugs/combinations on locomotion were assessed on naïve rats 1 h post-drug administration.

### 2.7. Chemicals

HU210, WIN55212 and STZ (streptozotocin) were purchased from Tocris Bioscience (Abingdon, UK). CFA (Complete Freund’s Adjuvant) was purchased from Sigma-Aldrich (Munich, Germany). Tramadol was purchased from The Arab Pharmaceutical Manufacturing Co. Ltd. (Amman, Jordan). Morphine was purchased from Hikma Pharmaceuticals LLC (Amman, Jordan). Unless otherwise indicated, drugs were initially dissolved in ethanol (100%) to form a stock solution, and then diluted in (3% tween 20 in saline).

### 2.8. Data Analysis

Latin square design was used in conducting behavioral experiments. Following the measurement of CFA and STZ-induced mechanical allodynia as paw withdrawal threshold (PWT) in grams, the antinociceptive effects of different drugs or combinations were quantified for each animal as % maximal possible effect (MPE). The following formula was used to quantify % MPE:

% MPE = (PWT after drug application − PWT before drug application)/(PWT before manipulation [CFA or STZ] − PWT before drug application) × 100.(1)

Two-way ANOVA analysis followed by Holm-Sidak post-hoc was used with treatment and time as the main factors. Furthermore, Dunnett’s post-hoc test was used when appropriate following one-way ANOVA test. Statistical analyses were conducted using Graph Pad statistical software (Prism 6, San Diego, CA, USA).

## 3. Results

### 3.1. Establishment of CFA and STZ-Induced Mechanical Allodynia

Intraplantar injection of CFA produced a noticeable local edema and redness after a few hours. mechanical PWT were significantly reduced 1 day after CFA injection. This reduction persisted for about three weeks (Supplementary Figure S1A). After STZ injection, in addition to changes in weight and blood glucose levels, hindpaw mechanical withdrawal thresholds were significantly decreased in the STZ-treated rats compared to the vehicle-treated rats, which indicates the development of apparent pain response (Supplementary Figure S1B).

### 3.2. Effects of Different Drugs or Combinations on Inflammatory Pain Model

**Figure 1:**
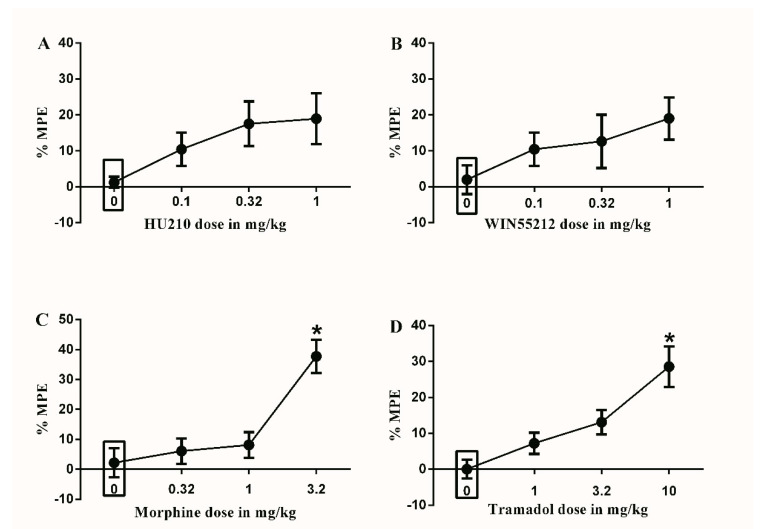
Effects of intraperitoneal injections of different drugs on Complete Freund’s Adjuvant (CFA)-induced mechanical allodynia. (**A**) Effects of intraperitoneal injections of HU210 (0.1, 0.32 and 1 mg/kg) or vehicle (3% tween 20 in saline) on CFA-induced changes in % maximal possible effect (MPE). One-way ANOVA revealed the following results: Main effect of treatment (F(3,28) = 2.339; *p* = 0.0949), (**B**) Effects of intraperitoneal injections of WIN55212 (0.1, 0.32 and 1 mg/kg) or vehicle (3% tween 20 in saline) on CFA-induced changes in % MPE. One-way ANOVA revealed the following results: Main effect of treatment (F(3,28) = 1.554; *p* = 0.2227), (**C**) Effects of intraperitoneal injections of morphine (0.32, 1 and 3.2 mg/kg) or vehicle (3% tween 20 in saline) on CFA-induced changes in % MPE. One-way ANOVA revealed the following results: Significant main effect of treatment (F(3,28) = 11.75; *p* < 0.0001), (**D**) Effects of intraperitoneal injections of tramadol (1, 3.2 and 10 mg/kg) or vehicle (3% tween 80 in saline) on CFA-induced changes in % MPE. One-way ANOVA revealed the following results: Significant main effect of treatment (F(3,28) = 9.916; *p* < 0.0001). Data are expressed as mean ± SEM of % MPE. One-way ANOVA test was used to analyze data followed by Dunnett’s post-hoc test, * *p* < 0.05, *n* = 8 rats per group. % MPE = (PWT after drug application − PWT before drug application)/(PWT before manipulation [CFA] − PWT before drug application) × 100. PWT indicates paw withdrawal threshold in grams. Rectangular boxes indicate vehicle treatment.

None of the used doses of HU210 or WIN55212 induced a significant effect on the % MPE compared to vehicle (Figure 1A,B). On the other hand, only the maximal doses of morphine (3.2 mg/kg) and tramadol (10 mg/kg) significantly increased the % MPE compared to vehicle (37.7 ± 5.9 vs. 2.2 ± 5.2 and 28.5 ± 6.1 vs. 0.1 ± 2.6, respectively, * *p* < 0.05, one-way ANOVA, *n* = 8, Figure 1C,D).

**Figure 2:**
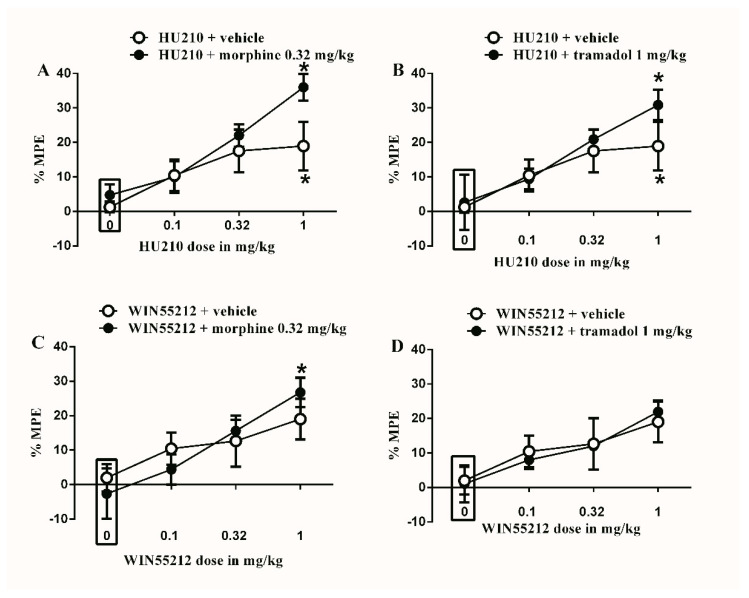
Effects of intraperitoneal injections of different drug combinations on CFA-induced mechanical allodynia. (**A**) Effects of intraperitoneal injections of HU210 (0.1, 0.32 and 1 mg/kg) plus the subtherapeutic dose of morphine (0.32 mg/kg) on CFA-induced changes in % MPE. Two-way ANOVA revealed the following results: Significant main effect of drug dose (F(3,48) = 9.492; *p* < 0.0001), main effect of combination (F(1,48) = 3.104; *p* = 0.0845), and main combination X drug dose interaction (F(3,48) = 1.186; *p* = 0.325), (**B**) Effects of intraperitoneal injections of HU210 (0.1, 0.32 and 1 mg/kg) plus the subtherapeutic dose of tramadol (1 mg/kg) on CFA-induced changes in % MPE. Two-way ANOVA revealed the following results: Significant main effect of drug dose (F(3,48) = 7.434; *p* = 0.0003), main effect of combination (F(1,48) = 1.105; *p* = 0.2984), and main combination X drug dose interaction (F(3,48) = 0.06326; *p* = 0.5775), (**C**) Effects of intraperitoneal injections of WIN55212 (0.1, 0.32 and 1 mg/kg) plus the subtherapeutic dose of morphine (0.32 mg/kg) on CFA-induced changes in % MPE. Two-way ANOVA revealed the following results: Significant main effect of drug dose (F(3,48) = 6.288; *p* = 0.0011), main effect of combination (F(1,48) = 3.2; *p* = 0.9955), and main combination X drug dose interaction (F(3,48) = 0.06806; *p* = 0.5682), (**D**) Effects of intraperitoneal injections of WIN55212 (0.1, 0.32 and 1 mg/kg) plus the subtherapeutic dose of tramadol (1 mg/kg) on CFA-induced changes in % MPE. Two-way ANOVA revealed the following results: Significant main effect of drug dose (F(3,48) = 4.764; *p* = 0.0055), main effect of combination (F(1,48) = 0.006004; *p* = 0.9396), and main combination X drug dose interaction (F(3,48) = 0.1010; *p* = 0.9591). Two-way ANOVA test was used to analyze data followed by Holm–Sidak post-hoc test. * indicates a difference that is significant compared with day 0 within the same rat group. Data represent mean ± SEM of 8 rats. % MPE = (PWT after drug application − PWT before drug application)/(PWT before manipulation [CFA] − PWT before drug application) × 100. PWT indicates paw withdrawal threshold in grams. Rectangular boxes indicate vehicle treatment.

The subtherapeutic doses of morphine (0.32 mg/kg) or tramadol (1 mg/kg) were co-administered with the different doses of HU210. The subtherapeutic doses of morphine (0.32 mg/kg) did not significantly enhance the antinociceptive effect of HU210 (Figure 2A). Similarly, the subtherapeutic dose of tramadol (1 mg/kg) did not significantly enhance the antinociceptive effect of HU210 (Figure 2B). Next, the subtherapeutic doses of morphine (0.32 mg/kg) or tramadol (1 mg/kg) were co-administered with the different doses of WIN55212. Morphine (0.32 mg/kg) did not significantly enhance the antinociceptive effect of WIN55212 (Figure 2C). Similarly, the subtherapeutic dose of tramadol (1 mg/kg) did not significantly enhance the antinociceptive effect of WIN55212 (Figure 2D).

### 3.3. Effects of Different Drugs or Combinations on Diabetic Neuropathy Pain Model

**Figure 3:**
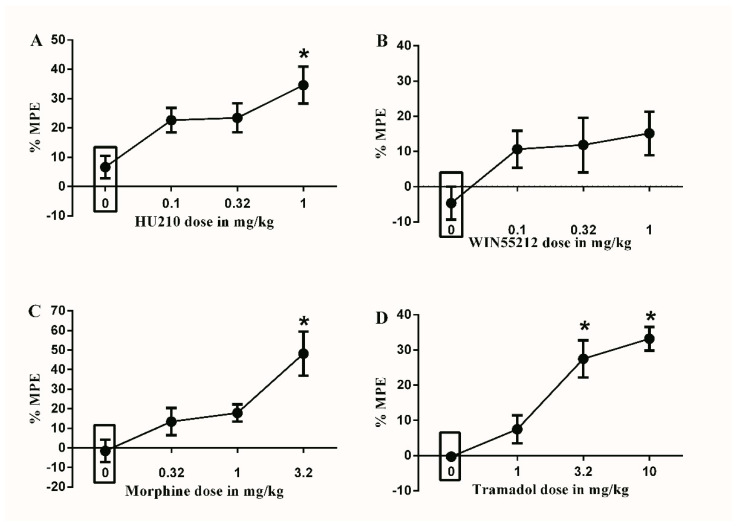
Effects of intraperitoneal injections of different drugs on STZ-induced mechanical allodynia. (**A**) Effects of intraperitoneal injections of HU210 (0.1, 0.32 and 1 mg/kg) or vehicle (3% tween 20 in saline) on STZ-induced changes in % MPE. One-way ANOVA revealed the following results: Significant main effect of treatment (F(3,28) = 5.474; *p* = 0.0043), (**B**) Effects of intraperitoneal injections of WIN55212 (0.1, 0.32 and 1 mg/kg) or vehicle (3% tween 20 in saline) on STZ-induced changes in % MPE. One-way ANOVA revealed the following results: Main effect of treatment (F(3,28) = 2.102; *p* = 0.1225). (**C**) Effects of intraperitoneal injections of morphine (0.32, 1 and 3.2 mg/kg) or vehicle (3% tween 20 in saline) on STZ-induced changes in % MPE. One-way ANOVA revealed the following results: Significant main effect of treatment (F(3,28) = 7.553; *p* = 0.0007), (**D**) Effects of intraperitoneal injections of tramadol (1, 3.2 and 10 mg/kg) or vehicle (3% tween 20 in saline) on STZ-induced changes in % MPE. One-way ANOVA revealed the following results: Significant main effect of treatment (F(3,28) = 18.59; *p* < 0.0001). Data are expressed as mean ± SEM of % MPE. One-way ANOVA test was used to analyze data followed by Dunnett’s post-hoc test, * *p* < 0.05, *n* = 8 rats per group. % MPE = (PWT after drug application − PWT before drug application)/(PWT before manipulation [STZ] − PWT before drug application) × 100. PWT indicates paw withdrawal threshold in grams. Rectangular boxes indicate vehicle treatment.

Only the maximal dose of HU210 (1 mg/kg) significantly increased the % MPE compared to vehicle (34.7 ± 4.8 vs. 6.2 ± 5.8, * *p* < 0.05, one-way ANOVA, *n* = 8, Figure 3A). On the other hand, none of the tested doses of WIN55212 produced a significant effect on the % MPE compared to vehicle (Figure 3B).

Only the maximal dose of morphine (3.2 mg/kg) significantly increased the % MPE compared to vehicle (48.1 ± 10.8 vs. 1.7 ± 3.8, * *p* < 0.05, one-way ANOVA, *n* = 8, Figure 3C). On the other hand, the maximal (10 mg/kg) and sub-maximal (3.2 mg/kg) doses of tramadol significantly increased the % MPE compared to vehicle (33.2 ± 2.5 vs. 0.2 ± 0.2 and 27.5 ± 5.7 vs. 0.2 ± 0.2, respectively, * *p* < 0.05, one-way ANOVA, *n* = 8, Figure 3D).

**Figure 4:**
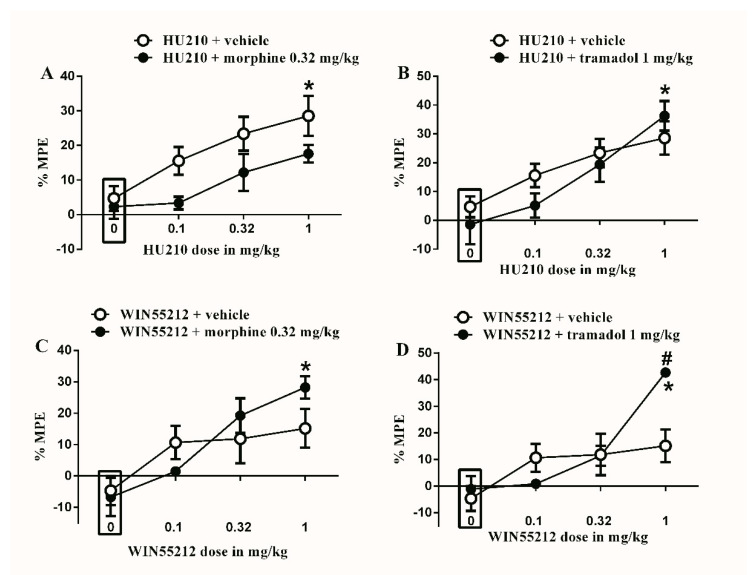
Effects of intraperitoneal injections of different drug combinations on STZ-induced mechanical allodynia. (**A**) Effects of intraperitoneal injections of HU210 (0.1, 0.32 and 1 mg/kg) plus the subtherapeutic dose of morphine (0.32 mg/kg) on STZ-induced changes in % MPE. Two-way ANOVA revealed the following results: Significant main effect of drug dose (F(3,48) = 7.855; *p* = 0.0002), Significant main effect of combination (F(1,48) = 8.748; *p* = 0.0048), and main combination X drug dose interaction (F(3,48) = 0.5447; *p* = 0.6541), (**B**) Effects of intraperitoneal injections of HU210 (0.1, 0.32 and 1 mg/kg) plus the subtherapeutic dose of tramadol (1 mg/kg) on STZ-induced changes in % MPE. Two-way ANOVA revealed the following results: Significant main effect of drug dose (F(3,48) = 13.61; *p* < 0.0001), main effect of combination (F(1,48) = 0.7796; *p* = 0.3817), and main combination X drug dose interaction (F(3,48) = 1.141; *p* = 0.3419), (**C**) Effects of intraperitoneal injections of WIN55212 (0.1, 0.32 and 1 mg/kg) plus the subtherapeutic dose of morphine (0.32 mg/kg) on STZ-induced changes in % MPE. Two-way ANOVA revealed the following results: Significant main effect of drug dose (F(3,48) = 8.758; *p* < 0.0001), main effect of combination (F(1,48) = 0.326; *p* = 0.5707), and main combination X drug dose interaction (F(3,48) = 1.493; *p* = 0.2284), (**D**) Effects of intraperitoneal injections of WIN55212 (0.1, 0.32 and 1 mg/kg) plus the subtherapeutic dose of tramadol (1 mg/kg) on STZ-induced changes in % MPE. Two-way ANOVA revealed the following results: Significant main effect of drug dose (F(3,48) = 12.55; *p* < 0.0001), main effect of combination (F(1,48) = 1.875; *p* = 0.1773), and Significant main combination X drug dose interaction (F(3,48) = 4.348; *p* = 0.0083). Two-way ANOVA test was used to analyze data followed by Holm–Sidak post-hoc test. # indicates a difference that is significant between the different rat groups. * indicates a difference that is significant compared with day 0 within the same rat group. Data represent mean ± SEM of 8 rats. % MPE = (PWT after drug application − PWT before drug application)/(PWT before manipulation [STZ] − PWT before drug application) × 100. PWT indicates paw withdrawal threshold in grams. Rectangular boxes indicate vehicle treatment.

The subtherapeutic doses of morphine (0.32 mg/kg) or tramadol (1 mg/kg) were co-administered with the different doses of HU210. An amount of 0.32 mg/kg morphine did not significantly enhance the antinociceptive effect of HU210 (Figure 4A). Similarly, the subtherapeutic dose of tramadol (1 mg/kg) did not significantly enhance the antinociceptive effect of HU210 (Figure 4B).

The subtherapeutic doses of morphine (0.32 mg/kg) or tramadol (1 mg/kg) were co-administered with the different doses of WIN55212. Morphine (0.32 mg/kg) did not significantly enhance the antinociceptive effect of WIN55212 (Figure 4C). In contrast, the subtherapeutic dose of tramadol (1 mg/kg) significantly enhances the antinociceptive effect of WIN55212 (42.7 ± 0.9 vs. 15.4 ± 4.2, * *p* < 0.05, two-way ANOVA, *n* = 8, Figure 4D).

### 3.4. Effects of Cannabinoids, Opioids or Their Combinations on Locomotion

**Figure 5:**
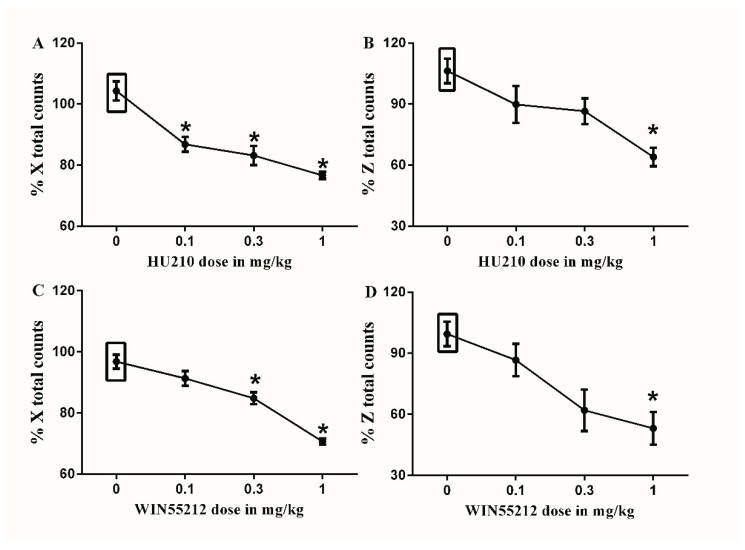
Effects of intraperitoneal injections of cannabinoids on locomotion. (**A**) Effects of intraperitoneal injections of HU210 (0.1, 0.32 and 1 mg/kg) or vehicle (3% tween 20 in saline) on % X total counts. One-way ANOVA revealed the following results: Significant main effect of treatment (F(3,20) = 20.62; *p* < 0.0001), (**B**) Effects of intraperitoneal injections of HU210 (0.1, 0.32 and 1 mg/kg) or vehicle (3% tween 20 in saline) on % Z total counts, One-way ANOVA revealed the following results: Significant main effect of treatment (F(3,20) = 6.758; *p* = 0.0025), (**C**) Effects of intraperitoneal injections of WIN55212 (0.1, 0.32 and 1 mg/kg) or vehicle (3% tween 20 in saline) on % X total counts. One-way ANOVA revealed the following results: Significant main effect of treatment (F(3,20) = 31.83; *p* < 0.0001), (**D**) Effects of intraperitoneal injections of WIN55212 (0.1, 0.32 and 1 mg/kg) or vehicle (3% tween 20 in saline) on % Z total counts. One-way ANOVA revealed the following results: Significant main effect of treatment (F(3,20) = 6.881; *p* = 0.0023), Data are expressed as mean ± SEM of % X and % Z total counts. One-way ANOVA test was used to analyze data followed by Dunnett’s post-hoc test, * *p* < 0.05, *n* = 8 rats per group. % X total counts = (X total counts after drug application/baseline X total counts) × 100. % Z total counts = (Z total counts after drug application/baseline Z total counts) × 100. Rectangular boxes indicate vehicle treatment.

All doses of HU210 significantly decreased the % X total counts compared to the vehicle-treated controls (76.5 ± 1.3 vs. 104.1 ± 3.4, 83.3 ± 3.4 vs. 104.1 ± 3.4 and 87.1 ± 2.7 vs. 104.1 ± 3.4, respectively, * *p* < 0.05, one-way ANOVA, Figure 5A). While only the maximal dose of HU210 (1 mg/kg) significantly decreased the % Z total counts compared to the vehicle-treated controls (64 ± 4.96 vs. 106. ± 6.6, * *p* < 0.05, one-way ANOVA, Figure 5B).

The maximal (1 mg/kg) and sub-maximal (0.32 mg/kg) doses of WIN55212 significantly decreased the % X total counts compared to the vehicle-treated controls (70.7 ± 1.0 vs. 96.8 ± 2.5 and 84.8 ± 2.2 vs. 96.8 ± 2.5, respectively, * *p* < 0.05, one-way ANOVA, Figure 5C). While only the maximal dose of WIN55212 (1 mg/kg) significantly decreased the % Z total counts compared to the vehicle-treated controls (53.5 ± 8.9 vs. 99.9 ± 6.5, * *p* < 0.05, one-way ANOVA, Figure 5D).

**Figure 6:**
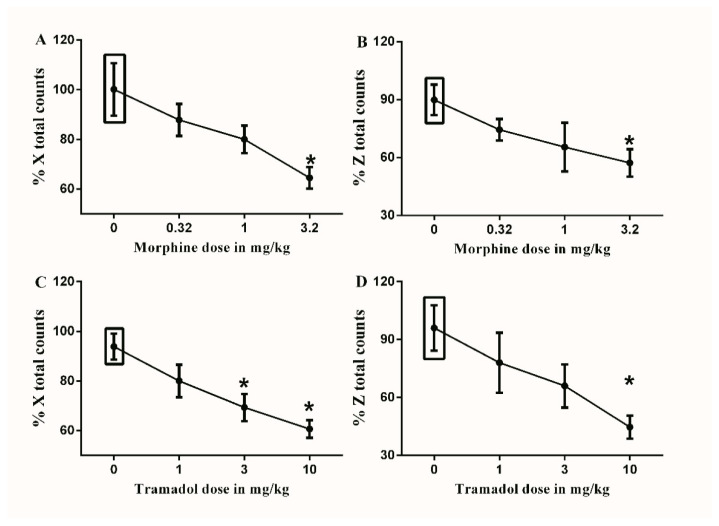
Effects of intraperitoneal injections of opioids on locomotion. (**A**) Effects of intraperitoneal injections of morphine (0.32, 1, and 3.2 mg/kg) or vehicle (3% tween 20 in saline) on % X total counts. One-way ANOVA revealed the following results: Significant main effect of treatment (F(3,20) = 4.406; *p* = 0.0156), (**B**) Effects of intraperitoneal injections of morphine (0.32, 1, and 3.2 mg/kg) or vehicle (3% tween 20 in saline) on % Z total counts. One-way ANOVA revealed the following results: Significant main effect of treatment (F(3,20) = 2.583; *p* = 0.0819), (**C**) Effects of intraperitoneal injections of tramadol (1, 3 and 10 mg/kg) or vehicle (3% tween 20 in saline) on % X total counts. One-way ANOVA revealed the following results: Significant main effect of treatment (F(3,20) = 7.232; *p* = 0.0018), (**D**) Effects of intraperitoneal injections of tramadol (1, 3 and 10 mg/kg) or vehicle (3% tween 20 in saline) on % Z total counts. One-way ANOVA revealed the following results: Significant main effect of treatment (F(3,20) = 4.972; *p* = 0.0097). Data are expressed as mean ± SEM of % X and % Z total counts. One-way ANOVA test was used to analyze data followed by Dunnett’s post-hoc test, * *p* < 0.05, *n* = 8 rats per group. % X total counts = (X total counts after drug application/baseline X total counts) × 100. % Z total counts = (Z total counts after drug application/baseline Z total counts) × 100. Rectangular boxes indicate vehicle treatment.

The maximal dose of morphine (3.2 mg/kg) significantly decreased both the % X total and % Z total counts compared to the vehicle-treated controls (65.5 ± 4.7 vs. 100.2 ± 11.7 and 57.3 ± 7.9 vs. 89.6 ± 7.9, respectively, * *p* < 0.05, one-way ANOVA, Figure 6A,B).

The maximal (10 mg/kg) and sub-maximal (3.2 mg/kg) doses of tramadol significantly decreased the % X total counts compared to the vehicle-treated controls (60.7 ± 3.9 vs. 93.7 ± 5.8 and 69.4 ± 6.0 vs. 93.7 ± 5.8, respectively, * *p* < 0.05, one-way ANOVA, Figure 6C). While only the maximal dose of tramadol (10 mg/kg) significantly decreased the % Z total counts compared to the vehicle-treated controls (44.7 ± 6.6 vs. 96.2 ± 13.0, * *p* < 0.05, one-way ANOVA, Figure 6D).

**Figure 7:**
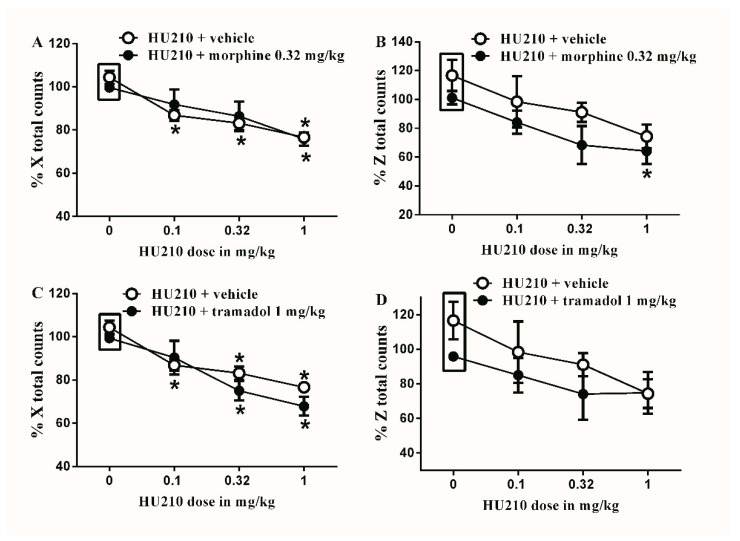
Effects of intraperitoneal injections of HU210 combined with morphine or tramadol on locomotion. (**A**) Effects of intraperitoneal injections of HU210 (0.1, 0.32 and 1 mg/kg) plus the subtherapeutic dose of morphine (0.32 mg/kg) on % X total counts. Two-way ANOVA revealed the following results: Significant main effect of drug dose (F(3,40) = 13.89; *p* < 0.0001), main effect of combination (F(1,40) = 0.05348; *p* = 0.08183), and main combination X drug dose interaction (F(3,40) = 0.5588; *p* = 0.6553), (**B**) Effects of intraperitoneal injections of HU210 (0.1, 0.32 and 1 mg/kg) plus the subtherapeutic dose of morphine (0.32 mg/kg) on % Z total counts. Two-way ANOVA revealed the following results: Significant main effect of drug dose (F(3,40) = 5.749; *p* = 0.0032), main effect of combination (F(1,40) = 4.788; *p* = 0.0347), and main combination X drug dose interaction (F(3,40) = 0.1388; *p* = 0.9362) (**C**) Effects of intraperitoneal injections of HU210 (0.1, 0.32 and 1 mg/kg) plus the subtherapeutic dose of tramadol (1 mg/kg) on % X total counts. Two-way ANOVA revealed the following results: Significant main effect of drug dose (F(3,40) = 20.48; *p* < 0.0001), main effect of combination (F(1,40) = 2.666; *p* = 0.1104), and main combination X drug dose interaction (F(3,40) = 1.001; *p* = 0.4023) (**D**) Effects of intraperitoneal injections of HU210 (0.1, 0.32 and 1 mg/kg) plus the subtherapeutic dose of tramadol (1 mg/kg) on % Z total counts. Two-way ANOVA revealed the following results: Significant main effect of drug dose (F(3,40) = 3.134; *p* = 0.0363), main effect of combination (F(1,40) = 2.674; *p* = 0.1101), and main combination X drug dose interaction (F(3,40) = 0.3628; *p* = 0.7764). Two-way ANOVA test was used to analyze data followed by Holm–Sidak post-hoc test. * indicates a difference that is significant compared with day 0 within the same rat group. Data represent mean ± SEM of 8 rats. % X total counts = (X total counts after drug application/baseline X total counts) × 100. % Z total counts = (Z total counts after drug application/baseline Z total counts) × 100. Rectangular boxes indicate vehicle treatment.

**Figure 8:**
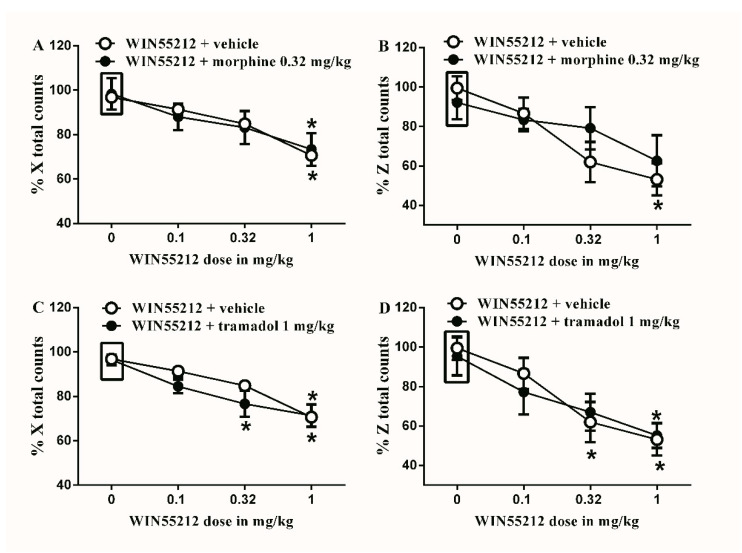
Effects of intraperitoneal injections of WIN55212 combined with morphine or tramadol on locomotion. (**A**) Effects of intraperitoneal injections of WIN55212 (0.1, 0.32 and 1 mg/kg) plus the subtherapeutic dose of morphine (0.32 mg/kg) on % X total counts. Two-way ANOVA revealed the following results: Significant main effect of drug dose (F(3,40) = 8.789; *p* = 0.0001), main effect of combination (F(1,40) = 0.0032; *p* = 0.9545), and main combination X drug dose interaction (F(3,40) = 0.1459; *p* = 0.9317), (**B**) Effects of intraperitoneal injections of WIN55212 (0.1, 0.32 and 1 mg/kg) plus the subtherapeutic dose of morphine (0.32 mg/kg) on % Z total counts. Two-way ANOVA revealed the following results: Significant main effect of drug dose (F(3,40) = 6.867; *p* = 0.0008), main effect of combination (F(1,40) = 0.4038; *p* = 0.5288), and main combination X drug dose interaction (F(3,40) = 0.8312; *p* = 0.4848), (**C**) Effects of intraperitoneal injections of WIN55212 (0.1, 0.32 and 1 mg/kg) plus the subtherapeutic dose of tramadol (1 mg/kg) on % X total counts. Two-way ANOVA revealed the following results: Significant main effect of drug dose (F(3,40) = 20.86; *p* < 0.0001), main effect of combination (F(1,40) = 2.367; *p* = 0.1318), and main combination X drug dose interaction (F(3,40) = 0.8829; *p* = 0.4582), (**D**) Effects of intraperitoneal injections of WIN55212 (0.1, 0.32 and 1 mg/kg) plus the subtherapeutic dose of tramadol (1 mg/kg) on % Z total counts. Two-way ANOVA revealed the following results: Significant main effect of drug dose (F(3,40) = 9.842; *p* < 0.0001), main effect of combination (F(1,40) = 0.07276; *p* = 0.7888), and main combination X drug dose interaction (F(3,40) = 0.2696; *p* = 0.8469). Two-way ANOVA test was used to analyze data followed by Holm–Sidak post-hoc test. * indicates a difference that is significant compared with day 0 within the same rat group. Data represent mean ± SEM of 8 rats. % X total counts = (X total counts after drug application/baseline X total counts) × 100. % Z total counts = (Z total counts after drug application/baseline Z total counts) × 100. Rectangular boxes indicate vehicle treatment.

The subtherapeutic doses of morphine (0.32 mg/kg) or tramadol (1 mg/kg) were co-administered with the different doses of HU210. Neither of the doses significantly alter HU210-induced locomotor impairment both in terms of % X total and % Z total counts (Figure 7). Similarly, the subtherapeutic doses of morphine (0.32 mg/kg) or tramadol (1 mg/kg) were co-administered with the different doses of WIN55212. Neither of the doses significantly alter WIN55212-induced locomotor impairment both in terms of % X total and % Z total counts (Figure 8).

## 4. Discussion

Of the tested combinations used in this study, only a subtherapeutic dose of tramadol increased the antinociceptive effect of WIN55212 in neuropathic pain (but not in inflammatory pain model) without any further reduction in locomotion activity. The current findings confirm previous observations showing that the antinociceptive effect of peripheral ∆9-THC was enhanced by the activation of the opioid system [16]. It is possible that the increased antinociceptive responses obtained with the combination of tramadol and WIN55212 might increase their individual antinociceptive effects through a multi-target mechanism of action. The findings in the present study provided evidence that WIN55212 in combination with tramadol produced a significant reduction in the nociceptive response; yet, the underlying mechanisms were not established. However, possible mechanisms might include pre- and post-junctional inhibition or stimulation, receptor co-localization with amplified signal transduction, and receptor stimulation by endogenous cannabinoid or/and opioid ligands [27,28]. Such interactions were of interest because of their therapeutic potential, especially since this combination did not produce any effects on the rat’s motility. Future studies are required to define the underlying mechanisms mediating interactions between tramadol and WIN55212. Investigating the analgesic activity of these combinations at various time points will enrich our understanding of how these novel drug combinations might modulate pain.

Tramadol exerted its antinociceptive effects by several suggested mechanisms, including activating opioid receptors, principally the μ type [29,30]. However, non-opioid mechanisms were also suggested, such as the inhibition of reuptake of noradrenaline and serotonin [29,30], or even activating the TRPV1 or imidazolinic receptors, which might be implied in the antinociceptive effect of tramadol [31]. The fact that tramadol is an inhibitor of the reuptake of noradrenaline and serotonin could could explain why tramadol increased the antinociceptive effect of WIN55212 in neuropathic pain (but not in inflammatory pain models). This issue might be supported by the fact that changes in serotoninergic and noradrenergic descending pain pathways during painful diabetic neuropathy have been reported in STZ-treated rats [32]. The concentration of different monoaminergic neurotransmitters (noradrenaline, dopamine, and serotonin) was found to be altered in the diabetic rat brain [33,34]. Furthermore, it is possible that these several suggested distinctive mechanisms for antinociceptive action of tramadol (a μ opioid agonist and monoamine reuptake-blocker), compared to morphine, explain the effect of a subtherapeutic dose of tramadol (but not the morphine) that increased the antinociceptive effect of WIN55212 in neuropathic pain.

Although the presence of a cross-talk between monoaminergic and CB2 receptors is yet to be explored, and considering that HU210 has higher affinity for CB1 receptors while WIN55212 has a higher selectivity for CB2 receptors [35], these facts could further explain why combining tramadol with HU210 was not as effective as combining it with WIN55212 in the model of neuropathic pain. Of note, HU210 is a high-affinity CB1 and CB2 receptor agonist [36]. CB1 receptors appear to mediate the in vivo effects of HU210 as evidenced by attenuation of those effects by CB1 receptor antagonists [37,38], as well as in CB1 receptor knockout mice [39]. Taken together, it is possible that the effect of a subtherapeutic dose of tramadol that increased the antinociceptive effect of WIN55212 (but not of HU210) in neuropathic pain ascribed to differences in the affinity for CB2 receptors between HU210 and WIN55212, suggesting a main potential contribution of CB2 receptors. This is in line with previous literature, in which the activation of spinal cannabinoid CB2 receptors inhibits neuropathic pain in streptozotocin-induced diabetic mice [40]. In fact, it is also possible that the antinociceptive effects of WIN55212 might be partially due to its anti-inflammatory effect through the downstream signaling of CB2 receptors [41]. Further research is needed to confirm these suggested explanatory mechanisms.

Although the mechanisms in which cannabinoids achieve their analgesic effects have not yet been fully understood, their actions are believed to be mediated by central (spinal and supra-spinal) and peripheral mechanisms of action [42,43]. In the present study, the synthetic cannabinoid HU210 did not significantly alleviate mechanical allodynia in inflammatory pain, while only the maximal dose (1 mg/kg) produced significant antinociceptive effect in the neuropathic pain model. Our results do not agree with previous work showing the efficacy of HU210 anti-allodynic effects in both models of inflammatory and neuropathic pain at relatively lower doses [44,45]. We attribute this to the different methodologies and pain models used. In this study, drugs were introduced at day 3 following CFA injection, while, in previous studies, drugs were administered 24 h post-CFA injection. This could suggest that the antinociceptive effects of HU210 are dependent at the time point of the model in which HU210 was injected, as there are currently no studies evaluating the time-course of its effects.

All the cannabinoid and opioid agonists used in this study dose-dependently reduced locomotor activity in the automated open field system. Both % Z and % X total counts decreased; reflecting that the overall locomotor activity, repetitive small-scale movements and rearing on hind paws are all affected. Regarding WIN55212 and HU210, these locomotion effects are mainly mediated by CB1 receptors [46,47]. Our results show limited dose separation between the anti-allodynic activity of each opioid and cannabinoid agonist and its side-effects on locomotion; indicating that the analgesic effects of these drugs are restricted by their narrow therapeutic window. This is consistent with previous studies exploring the effects of opioids, morphine and tramadol [48,49], and cannabinoids, HU210 and WIN55212 [44,50], on motor activity. In this respect, combination therapies with therapeutic or subtherapeutic doses of cannabinoid and opioid agonists may offer an alternative way to avoid these unwanted motor disturbances.

In conclusion, these findings further support the notion of interaction between the cannabinoid receptor ligands and opioids for pain management, and suggest that cannabinoid receptor ligands if combined with the suitable opioid may be more effective in clinical intervention. Combinations of cannabinoids and opioids, if effective, could have an important clinical value since this would allow to decrease the dosage of opioids that, in turn, could lead to a decrease in their abuse liability. In this context, opioid and cannabinoid neuromodulatory system may emerge as novel pathway to control pain. According to our data, the fact that cannabinoid-mediated antinociception can be more efficacious through simultaneous activation of the opioid system is therapeutically promising. Developing new analgesics that might simultaneously activate both cannabinoid and opioid receptors may be of great clinical value.

## Figures and Tables

**Table 1:** Latin square design showing the injection of each tested drug in days 3–9 post-CFA injection. HU210 (dose 1 = 0.1 mg/kg, dose 2 = 0.32 mg/kg and dose 3 = 1 mg/kg). WIN55212 (dose 1 = 0.1 mg/kg, dose 2 = 0.32 mg/kg and dose 3 = 1 mg/kg). Morphine (dose 1 = 0.32 mg/kg, dose 2 = 1 mg/kg and dose 3 = 3.2 mg/kg). Tramadol (dose 1 = 1 mg/kg, dose 2 = 32 mg/kg and dose 3 = 10 mg/kg).

	Days Post-CFA Injection			
	Day 3	Day 5	Day 7	Day 9
Rats 1 and 2	Vehicle	Dose 1	Dose 2	Dose 3
Rats 3 and 4	Dose 3	Vehicle	Dose 1	Dose 2
Rats 5 and 6	Dose 2	Dose 3	Vehicle	Dose 1
Rats 7 and 8	Dose 1	Dose 2	Dose 3	Vehicle

**Table 2:** Latin square design showing the injection of each tested drug in days 1–7 in week 4 post-streptozotocin (STZ) injection. HU210 (dose 1 = 0.1 mg/kg, dose 2 = 0.32 mg/kg and dose 3 = 1 mg/kg). WIN55212 (dose 1 = 0.1 mg/kg, dose 2 = 0.32 mg/kg and dose 3 = 1 mg/kg). Morphine (dose 1 = 0.32 mg/kg, dose 2 = 1 mg/kg and dose 3 = 3.2 mg/kg). Tramadol (dose 1 = 1 mg/kg, dose 2 = 32 mg/kg and dose 3 = 10 mg/kg).

	Days in Week 4 Post-STZ Injection			
	Day 1	Day 3	Day 5	Day 7
Rats 1 and 2	Vehicle	Dose 1	Dose 2	Dose 3
Rats 3 and 4	Dose 3	Vehicle	Dose 1	Dose 2
Rats 5 and 6	Dose 2	Dose 3	Vehicle	Dose 1
Rats 7 and 8	Dose 1	Dose 2	Dose 3	Vehicle

## Supplementary Materials

The following are available online at https://www.mdpi.com/2076-3425/10/8/523/s1, Figure S1: Reduction of paw withdrawal threshold (PWT) following injection of Complete Freund’s adjuvant (CFA) or streptozotocin (STZ).
