# Supplementary material for: Analgesic Effects and Impairment in Locomotor Activity Induced by Cannabinoid/Opioid Combinations in Rat Models of Chronic Pain
Source: Brain Sci. 2020 Aug 6;10(8):523. doi: 10.3390/brainsci10080523 (PMC7547378; doi:10.3390/brainsci10080523)
Supplement: Supplementary file 1 [file brainsci-10-00523-s001.pdf]

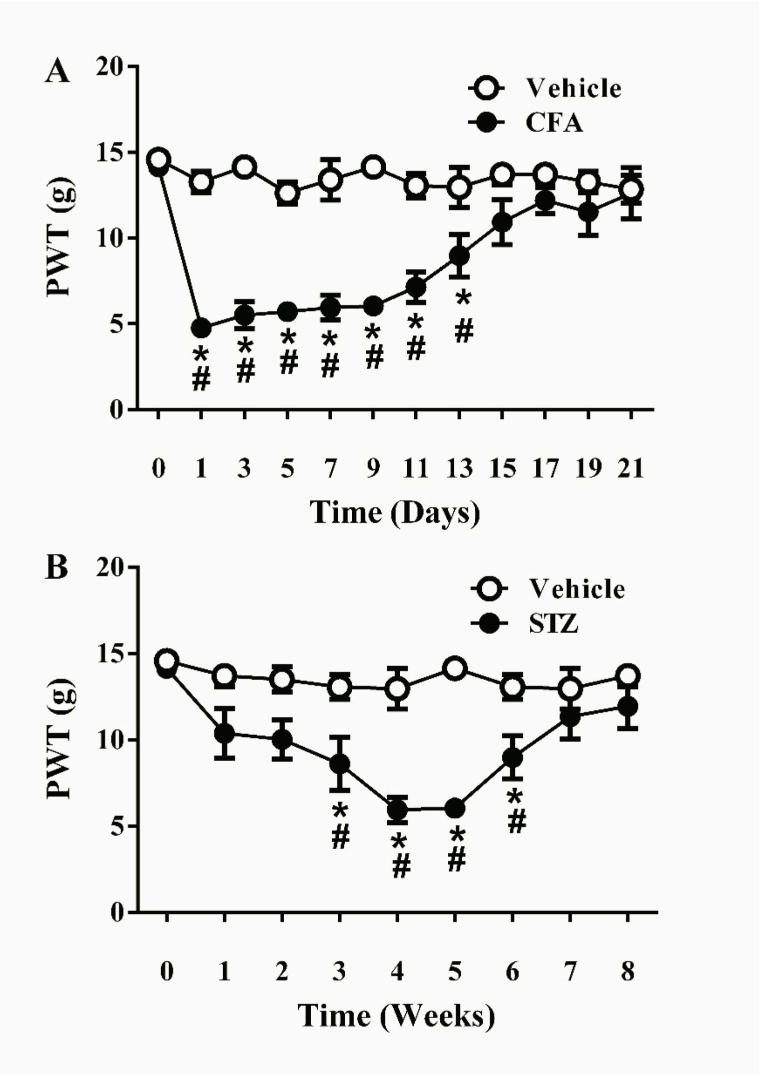

Figure S1. Reduction of paw withdrawal threshold (PWT) following injection of Complete Freund's adjuvant (CFA) or streptozotocin (STZ), **A.** Effects of intraplantar injection of CFA on Mechanical PWT. PWT was significantly decreased in CFA-treated rats compared to vehicle-treated rats. Two-way ANOVA revealed the following results: significant main effect of treatment [$F(1,144) = 182.8$; $p < 0.0001$], significant main effect of time [$F(11,144) = 8.5$; $p < 0.0001$], and significant main treatment X time interaction [$F(11,144) = 7.378$; $p < 0.0001$]. # indicate significant difference between groups. * indicates significant difference vs. day 0 within group. All data represent mean ± SEM of 8 rats. (PWT = paw withdrawal threshold in grams), **B.** Effects of intraperitoneal injection of STZ on Mechanical PWT. PWT was significantly decreased in STZ-treated rats compared with vehicle-treated rats. Two-way ANOVA revealed the following results: significant main effect of treatment [$F(1,108) = 67.87$; $p < 0.0001$], significant main effect of time [$F(8,108) = 4.547$; $p < 0.0001$], and significant main treatment X time interaction [$F(8,108) = 3.254$; $p < 0.0001$]. # indicate significant difference between groups. * indicates significant difference vs. day 0 within group. All data represent mean ± SEM of 7 rats. (PWT = paw withdrawal threshold in grams).
